# Effect of betamethasone injection into the pterygomandibular space on the neurosensory disturbance after bilateral sagittal split ramus osteotomy: a pilot study

**DOI:** 10.1186/s40001-023-01615-4

**Published:** 2024-02-01

**Authors:** Shehab Ahmed Hamad, Khurshid A. Kheder Khrwatany, Mustafa Rasul Mohammed, Saeed Hameed Tutmayi

**Affiliations:** 1 Kurdistan Higher Council of Medical Specialties, Erbil, Iraq; 2 Department of Oral and Maxillofacial Surgery, College of Dentistry, Hawler Medical University, Erbil, Iraq; 3 Department of Dental Assistant, Medical Institute, Erbil Polytechnic University, Erbil, Iraq

**Keywords:** Betamethasone, BSSO, Neurosensory disturbance, Inferior alveolar nerve

## Abstract

**Aim:**

The aim of this study was to evaluate the effect of local betamethasone injection into the pterygomandibular space on postoperative neurosensory deficits.

**Materials and methods:**

A prospective controlled clinical study was conducted on 16 patients (6 male, 10 female; mean age, 24.95 ± 9.22 years) who underwent bilateral sagittal ramus osteotomy for mandibular discrepancies. One side of each patient’s mandible was randomly selected as the control side, and the opposite side as the experimental side. On the experimental side, a solution of betamethasone (6 mg/1 ml) was injected into the pterygomandibular space after the completion of wound closure. Neurosensory tests, including light touch, two-point discrimination, direction of movement, thermal sensitivity, and pin-prick discrimination, were performed. The follow-up period ranged between 6 and 12 months, according to the particular sensory test. The Fisher exact test was used to analyse the data.

**Results:**

The light touch sensation was abnormal in 75% of the control side and 31% of the study side, with the difference being significant (*p* = 0.03). However, at 6 months, all the study cases regained touch sensation, compared to 69% of the control side. No significant difference in direction movement discrimination was seen; however, at 3 months, the study side showed significantly less direction sensation (19%) compared to the control side (56%) (*p* = 0.02). There was no significant difference in the two-point discrimination; however, at 3 months, the study side had a significantly less abnormal two-point sensation (13%) than the control side (56%) (*p* = 0.02). In addition, no significant difference was noted in thermal sensitivity or pin-prick sensation.

**Conclusion:**

Betamethasone injection into the pterygomandibular space reduces neurosensory disturbances after bilateral sagittal split ramus osteotomies nd leads to faster recovery of sensations.

## Introduction

Bilateral sagittal split ramus osteotomy (BSSO) is the most commonly performed mandibular orthognathic surgical procedure to relocate the body of the mandible to correct prognathism, retrognathism, or asymmetry. Neurosensory deficit (NSD) of the inferior alveolar nerve is one of the most common complications of BSSO [1]. The incidence of NSD immediately after BSSO ranges between 9 and 85% of patients [2]. However, other studies claim that 100% of patients develop this complication [3]. The prevalence of NSD one or two years after BSSO ranges from 0% up to 85% [3]. Numerous factors, including the diversity of nerve function evaluations, difference in follow-up times between studies, and assessor expertise, have been implicated in these variations [4].

Paresthesia, dysesthesia (burning, stinging, or stabbing sensations), sensory impairments, allodynia, or hyperesthesia are some of the reported symptoms associated with damaged nerves [5]. Both paresthesia and hypoesthesia are frequently reported [4]. NSD may have an impact on patients' daily lives and can cause social and psychological problems due to hyperesthesia and its detrimental effects on eating, drinking, speaking, and social interaction [6]. The inferior alveolar nerve may be compressed by bone fragments, damaged directly by mechanical stimulation of the nerve, or indirectly by surgical instruments or by the direction of movement of the distal bone fragments [7].

Many factors can affect NSD after BSSO, including patient age and sex, the extent and direction of movement of the distal segment, mandible cutting devices, the split pattern, the degree of nerve exposure and manipulation, the proximity of the mandibular canal to the buccal cortical plate, the method of fixation, the surgeon’s skill, and the method and timing of neurosensory testing [8].

The reflection of soft tissue and compression of the nerve on the medial side of the mandibular ramus by protective retractors are two additional significant factors linked to postoperative NSD. After subperiosteal dissection on the medial aspect of the mandibular ramus, it is crucial to place a suitable retractor, such as a channel retractor, directly above the lingula to protect the IAN and facilitate the medial horizontal bone cut. The channel retractor, while improving vision in this limited surgical field, is likely to overstretch the IAN during retraction to improve vision [9].

To minimize pain, swelling, trismus, and neurosensory disturbance, supportive medications have been recommended for patients undergoing operations like orthognathic surgery, impacted tooth surgery, and dental implantology. When given not later than one week after surgery, steroids have the ability to hasten the healing of sensory dysfunctions, according to Seo et al. [10].

Few studies have evaluated the effect of local and systemic corticosteroids on NSD after BSSO. The aim of this study was to investigate the effect of betamethasone injection into the pterygomandibular space on the NSD of the inferior alveolar nerve after BSSO.

## Materials and methods

### Study design and sample

**Fig. 1:**
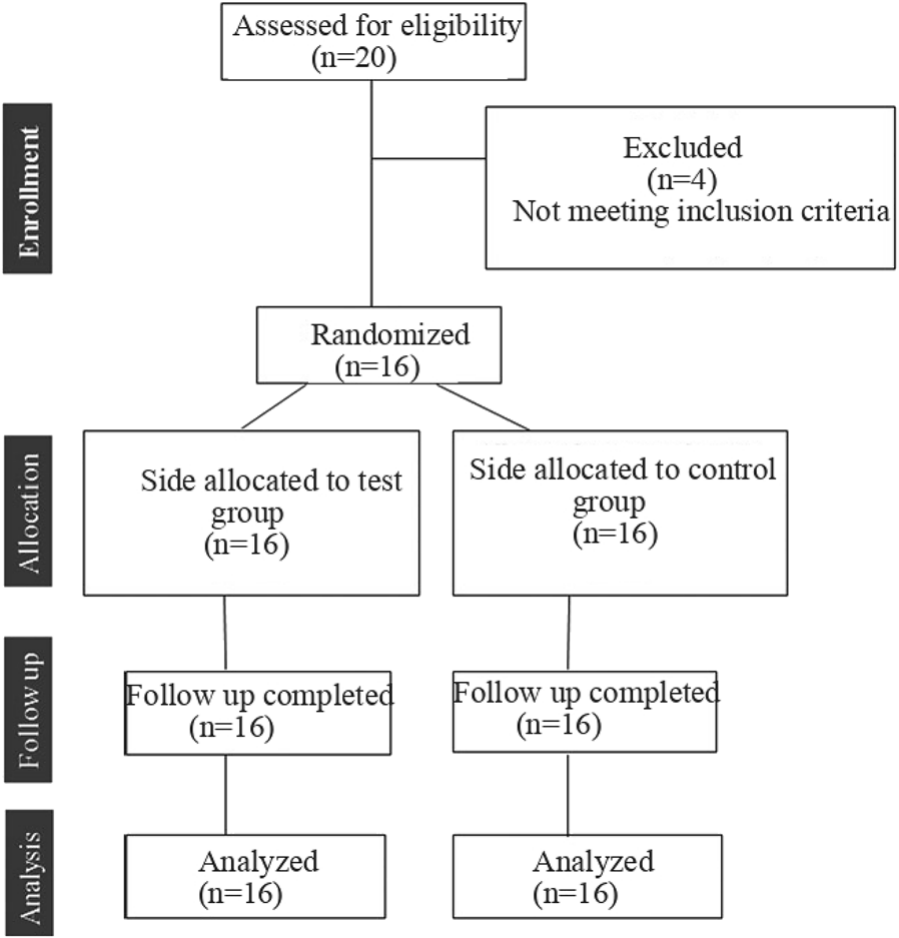
Consort flowchart diagram of the clinical trial

A prospective, randomized, placebo-controlled, double-blind study was conducted on patients who underwent bilateral sagittal split ramus osteotomy, with or without Le Fort I osteotomies, at CMC Hospital in Erbil, Iraq, from 2015 to 2022. The inclusion criteria were age less than 40 years and the absence of systemic diseases. Patients who underwent concomitant genioplasty, extraction of impacted third molars, and unfavourable splits were excluded. Patients with a previous history of mandibular trauma and those who used drugs were also excluded (Fig. 1).

The study adhered to the principles of the Declaration of Helsinki of 1975 (revised 2018) and received approval from the ethical committee board. All participants willingly agreed to take part in the study and provided by signing a consent form.

### Randomization

The BSSO sides were randomly assigned to two groups. The experimental side received a betamethasone local injection into the pterygomandibular space, while the control side received a sterile distilled water injection. The allocation of sides to receive either the experimental or sham solution was done using a simple randomization method involving a coin toss.

### Surgical procedures

All BSSO surgeries were performed by the same surgeon (S.A.H), following the Obwegeser/Dal˗Pont procedure with Hunsuck modification. After wound closure, the experimental side received a 1 ml (6 mg) injection of betamethasone (CKOÇAK FARMA, Turkey) into the pterygomandibular space. Each millilitre contained 3.0 mg of betamethasone acetate and 3.0 mg of betamethasone sodium phosphate. The control side received an equivalent volume of sterile distilled water injected into the pterygomandibular space. To maintain the blinding of the study, the solution was administered by a surgeon who was not directly involved in the surgery or postoperative visits.

After the surgical procedure, patients were provided with analgesics (ibuprofen or paracetamol) for as long as they found them beneficial. Additional pain medication during the immediate postoperative recovery, when needed, included codeine phosphate or tramadol. Patients also received antibiotics until postoperative days 7–10, typically penicillin or a first-generation cephalosporin. In the case of a penicillin allergy, clindamycin was prescribed.

### Study variables

To assess the sensory capacity of the inferior alveolar nerve, five neurosensory tests were conducted: touch (static light touch and direction of movement discrimination), temperature sensitivity, two-point discrimination, and the pin-prick test [11]. The tests were performed at the vermilion border of the lower lip (1 cm medial to the angle of the mouth) before surgery and at 1 week, 1 month, 3 months, 6 months, 9 months, and 12 months after surgery. All subjects underwent testing in a semi-reclined position with their eyes closed in a room at room temperature, free from any audio or visual disturbances. The same investigator performed the neurosensory tests on all patients in the same order. Each instrument's use was evaluated on a skin area with normal sensitivity before each neurosensory test. For every two or three stimuli that the patient was unable to identify, additional stimuli were applied to a skin location with normal sensitivity, establishing a baseline for a typical cutaneous feeling.

1. Static light touch: This test involved applying a piece of cotton, and the patient was instructed to raise their hand when they felt the sensation of touch.
2. Direction of movement discrimination: A piece of cotton was swiped 10 times for a distance of 1 cm in a right-to-left or left-to-right direction. When the correct response was 9 out of 10 (90%) or higher, the result was considered normal.
3. Static two-point discrimination: This test utilized a sharp millimetre calliper. It began with the points essentially touching, allowing the patient to discriminate only one point. Then, the calliper points were progressively opened in 1 mm increments until the patient could discriminate between two separate points of contact. There must be no skin blanching, as it indicates too much pressure being applied. The separation ranged from 1 to 20 mm. The test was considered abnormal when the patient perceived two points 3 mm farther apart.
4. Thermal sensitivity test: This test was conducted using two small glass tubes containing cold (15 to 20 ℃) or hot (40 to 45 ℃) water. The test area was randomly touched 10 times with the test tube and the patient had to decide whether the stimulus was hot or cold. The results of the test were deemed normal when equal to or above 90% of the answers were correct.
5. Pin-prick nociception test: This test was conducted by applying needles with a weight ranging between 0.5 and 15 g. If the weight of the needle was 1 g greater than that of the lightest needle at which the patients felt sharpness during the preoperative period, the test was considered abnormal.

### Statistical analysis

The data were analysed using SPSS version 28 computer software (IBM Corp., Armonk, NY, USA). Fisher’s exact test was used to compare differences in neurosensory test results between the two groups at five time points after surgery. The level of significance was considered at a *p*-value ≤ 0.05.

## Results

The study included 16 patients (6 male and 10 female), with a mean (SD) age of 24.95 (9.22) years, ranging from 17 to 40 years). All patients completed the follow-up neurosensory testing visits.

Light touch sensation was lost in 13 (81%) cases on the study side and 15 (93%) cases on the control side, with no significant difference noted (*p* = 0.599). However, at 6 months, all patients on the study side regained sensation, while five (31%) cases on the control side still had abnormal sensation, and this difference was statistically significant (*p* = 0.043). By 9 months, no patients on the study side experienced abnormal sensation (Table 1).

**Table 1:** Light touch test of the study and controlled sides

Schedule of light touch test	Control side, no. (%)		Study side, no. (%)		*p*-value
	Normal	Abnormal	Normal	Abnormal	
Preoperative	16 (100)	0 (00)	16 (100)	0 (00)	1.00
1 day	1(6.25)	15 (93.75)	3 (18.75)	13 (81.25)	0.599
1 month	4 (25)	12 (75)	11(68.75)	5 (31.25)	0.032*
3 months	9 (56.25)	7 (43.75)	14 (87.5)	2 (12.5)	0.113
6 months	11(68.75)	5 (31.25)	16 (100)	0 (00)	0.043*
9 months	16 (100)	0 (00)	16 (100)	0 (00)	1.00

Direction of movement discrimination was lost in 15 (93%) cases on the study side and in all cases on the control side. By 6 months postoperatively, all the study sides regained sensation, while the control sides full recovered by 9 months (Table 2).

**Table 2:** Direction of movement discrimination test of the study and control sides

Schedule of direction movement discrimination	Control side, no. (%)		Study side, no. (%)		*p*-value
	Normal	Abnormal	Normal	Abnormal	
Preoperative	16 (100)	0 (00)	16 (100)	0 (00)	1.00
1 day	0 (00)	16 (00)	1 (6.25)	15 (93.75)	1.00
1 month	3 (18.75)	13 (81.25)	8 (50)	8 (50)	0.135
3 months	7(43.75)	9 (56.25)	13 (81.25)	3 (18.75)	0.023*
6 months	12 (75)	4 (25)	16 (100)	0 (00)	0.101
9 months	16 (00)	0 (00)	16 (00)	0 (00)	1.00

Static two-point discrimination was abnormal in 12 (75%) cases on the study side and 14 (87.5%) cases on the control side, with no significant difference noted (*p* = 0.653). At 9 months, all study sides regained sensation, while three (18%) cases on the control side still had abnormal sensations. These sensations remained unchanged at 9 months, but fully recovered at 12 months (Table 3).

**Table 3:** Two-point discrimination test of the study and control sides

Schedule of two-point discrimination test	Control side, no. (%)		Study side, no. (%)		*p*-value
	Normal	Abnormal	Normal	Abnormal	
Preoperative	16 (100)	0 (00)	16 (100)	0 (00)	1.00
1 day	2 (12.5)	14 (87.5)	4 (25)	12 (75)	0.653
1 month	5 (31.25)	11 (68.75)	8 (50)	8 (50)	0.472
3 months	7 (43.75)	9 (56.25)	14 (87.5)	2 (12.5)	0.023^*^
6 months	13 (81.25)	3 (18.75)	14 (87.5)	2 (12.5)	1.00
9 months	13 (81.25)	3 (18.75)	16 (100)	0 (00)	1.00
12 months	16 (100)	0 (00)	16 (100)	0 (00)	1.00

The thermal sensitivity test was abnormal in all control sides and in 13 (81%) cases on the study sides, with no significant difference (*p* = 1.00). By 3 months, all cases on the study side regained sensation, whereas only 12 (75%) cases on the control side did so. The remaining four (25%) cases regained the thermal sensation at 6 months post-surgery (Table 4).

**Table 4:** Thermal sensitivity test of the study and control sides

Schedule of thermal sensitivity test	Control side, no. (%)		Study side, no. (%)		*p*-value
	Normal	Abnormal	Normal	Abnormal	
Preoperative	16 (100)	0 (00)	16 (100)	0 (00)	1.00
1 day	0 (00)	16 (100)	3 (18.75)	13 (81.25)	0.225
1 month	10 (62.5)	6 (37.5)	14 (87.5)	2 (12.5)	0.220
3 months	12 (75)	4 (25)	16 (100)	0 (00)	0.101
6 months	16 (100)	0 (00)	16 (100)	0 (00)	1.00

The pin-prick test was abnormal in 12 (75%) cases on both sides; however, both sides showed complete recovery of sensation at 6 months (Table 5).

**Table 5:** Pin-prick test of the study and control sides

Schedule of pin-prick test	Control side, no. (%)		Study side, no. (%)		*p*-value
	Normal	Abnormal	Normal	Abnormal	
Preoperative	16 (100)	0 (00)	16 (100)	0 (00)	1
1 day	4 (25)	12 (75)	4 (25)	12 (75)	1
1 month	12 (75)	4 (25)	13 (81.25)	3 (18.75)	1
3 months	14 (87.5)	2 (12.5)	15 (93.75)	1 (6.25)	1
6 months	16 (100)	0 (00)	16 (100)	0 (100)	1

## Discussion

This prospective controlled study aimed to investigate whether the NSD of the inferior alveolar nerve after sagittal split ramus osteotomy would be affected by the local injection of betamethasone into the pterygomandibular space. In this study, it was hypothesized that local betamethasone injection would lessen the NSD after BSSO. The results of the study supported this hypothesis, indicating that the betamethasone-injected sides exhibited less NSD than the control sides.

NSD of the inferior alveolar nerve, a concern throughout all stages of surgery, is the main issue in mandibular osteotomies, especially BSSO [12]. The inferior alveolar nerve follows the course of the osteotomy during BSSO, increasing the likelihood of nerve damage. The deficit may manifest as numbness or unusual sensations in the gingiva, teeth, chin, and lower lip. The majority of neurosensory defects identified during the postoperative phase are reversible [13]. However, neurosensory disturbances persisting even a year after a mandibular osteotomy should be considered more severe and permanent [14]. Consequently, only patients with at least 12 months of postoperative follow-up were analysed in this study.

Numerous factors, such as advanced age, female gender, nerve exposure and manipulation, direction of mandibular movement, and screw fixation, have been associated with inferior alveolar nerve NSD. Notably, older age is significantly correlated with postoperative NSD, attributed to axonal atrophy and a reduced ability to heal damaged nerves in older people [15]. Additionally, compared to male patients, female patients experience more postoperative NSD [16].

In this split-mouth, randomized clinical trial, each participant was compared with themselves, eliminating the need to evaluate age or sex as variables in this study. Moreover, NSD and greater mandibular advancement have been found to be significantly correlated [17]. In contrast to bicortical screw fixation, it has been reported that monocortical screw fixation results in a significantly lower incidence of neurosensory deficits, and these deficits recover completely by the end of the 12th month [18].

In a study aimed at predicting neurosensory alterations after SSRO, Kuroyanagi and Shimozato [9] reported that the development of NSD is associated with surgical exposure on the medial side of the mandibular ramus and subsequent manipulation of the IAN in that region. In the current study, the injection of betamethasone into the pterygomandibular space resulted in less NSD, likely through the reduction of inflammation and oedema around the nerve.

Pourdanesh et al. [19] demonstrated that the application of dexamethasone to the inferior alveolar nerve during BSSO had no preventive effects on NSD, attributing it to the washing effect of irrigation during surgery. The antiinflammatory effect of local betamethasone after spinal root decompression in cats was seen by Wong and Tan [20], noting the relative absence of cytokine differentiation antigens 4- and 5-labelled lymphocytes at the compression site in the steroid-treated group. Sencar et al. [21] investigated the antiinflammatory effect of betamethasone on crushed sciatic nerves in rats and found that combing the nerve growth factor with betamethasone resulted in rapid functional recovery. Al-Bishri et al. [22] identified a beneficial effect of betamethasone on the recovery of damaged sciatic nerves in rats, as reflected in the recruitment of macrophages and the expression of the p75 nerve growth factor receptor.

The limitation of this study is the small sample size and the effect of other factors on the incidence of nerve injury, such as the lingual split pattern, the presence of impacted lower third molars, and the exposure and manipulation of the inferior alveolar nerve during surgery. Hamad [23] found that betamethasone injection into the pterygomandibular space significantly reduced the inflammatory sequelae of impacted lower third molar surgery.

## Conclusion

The local injection of betamethasone into the pterygomandibular space significantly reduces neurosensory deficits after bilateral sagittal split osteotomies and promotes a faster recovery of nerve functions.

## Data Availability

The data are available on request by emailing the corresponding author Shehab Ahmed Hamad, at shehab.ahmed@khcms.edu.krd.
